# Simulated root canals preparation time, comparing ProTaper Next and WaveOne Gold systems, performed by an undergraduate student

**DOI:** 10.4317/jced.56981

**Published:** 2020-08-01

**Authors:** Inês Conceição, Inês Ferreira, Ana-Cristina Braga, Irene Pina-Vaz

**Affiliations:** 1 DMD, MDent, Faculty of Dental Medicine of University of Porto, Porto, Portugal; 2 PhD, Department of Production and Systems, ALGORITMI Center, University of Minho, Portugal; 3 DMD, PhD, Faculty of Dental Medicine of University of Porto, Porto, Portugal

## Abstract

**Background:**

The aim of this study was to compare the WaveOne Gold and ProTaper Next systems regarding the time spent preparing simulated canals performed by an inexperienced student.

**Material and Methods:**

0 simulated L-shaped canals were randomly divided into two groups (n = 40) and numbered in order of instrumentation. Canals were instrumented with WaveOne Gold (group 1) and ProTaper Next (group 2) systems. The effective instrumentation time and the number of instrumentation cycles were recorded. All procedures were performed by the same operator. Statistical analysis was obtained by the Mann - Whitney, Kruskal - Wallis test with significance of *p*<0.05.

**Results:**

There were no statistically significant differences regarding the mean instrumentation time between the two instrumentation systems. The instrumentation time decreased over the experimental period, regardless of the technique used.

**Conclusions:**

Through a short learning curve, an inexperienced operator can prepare simulated canals in a very predictable time. Time spent was similar in a multi-file instrument system (ProTaper Next) and a single-file system (WaveOne Gold).

** Key words:**Dental education, endodontics, preclinical, root canal preparation, undergraduate.

## Introduction

Biomechanical preparation is one of the most important phase for a successful endodontic treatment. This step aims to eliminate infected, necrotic or inflamed root canal tissue, create smooth walls that facilitate irrigation and obturation while maintaining the original canal shape (1).

The introduction of rotary nickel titanium (NiTi) instrumentation provided a faster and safer approach, with a lower risk of procedural errors compared to hand instrumentation (2,3).

Over the years new instrumentation systems have been developed and improved allowing us to overcome the difficulties of root canal preparation, simplify procedures and reduce instrumentation time (4). The new generation of NiTi rotary systems includes, among others, ProTaper Next (PTN) and WaveOne Gold (WOG). One of the most salient advantages of the reciprocating system is the ability to do all the preparation simply and quickly using a single file (5).

ProTaper Next (Dentsply Maillefer, Ballaigues, Switzerland) is a system made of a NiTi alloy called M-Wire and includes a series of files with a progressive apical calibre. ProTaper Next (PTN) files have a rectangular cross section and a continuous asymmetrical rotational movement which, according to the manufacturer, by reducing the contact points with the canal walls reduces the cyclic fatigue of the instrument.

WaveOne Gold (Dentsply Maillefer, Ballaigues, Switzerland) is a single-file reciprocating system with a rectangular cross section like PTN. The files are made of NiTi Gold alloy providing better flexibility and resistance to fracture.

Natural teeth and simulated canals are often used to study and compare different instrumentation systems (2,6). These simulated canals are recognized as valid study models allowing the standardization of experimental conditions by reducing the variability introduced through the different anatomical characteristics of natural teeth (7). However, extrapolation of these results to clinical situations should be done cautiously.

The aim of this study was to compare two rotary systems (WaveOne Gold and ProTaper Next) regarding the time spent preparing simulated canals, performed by a student without previous experience.

## Material and Methods

80 clear resin blocks with an L-shaped canal (Endo Training-Bloc-L, Dentsply Maillefer) were used. The length of the canal was determined by inserting a 10K file until the tip became visible on the apical foramen to the microscope (OPMI Pico, Carl Zeiss, Germany, 12.5X magnification). The working length (WL) was defined as 14mm for all canals.

The only operator was a final year dental student who had no experience with the use of the rotary instrumentation system. Its curriculum experience was exclusively with manual instrumentation with stainless steel files (stepback technique). Before the study the student received a brief training, which included written instructions, watching videos clips and training on a single clear simulated canal for each technique.

Patency of the canals was checked by advancing a size #10 K-file through the apical foramen. The glide path was then created using the ProGlider file (Dentsply Maillefer) at the full WL. Between each file, the canal was irrigated with 96% alcohol using a 27G syringe and permeabilized with the #10 K-file.

After that the canals were randomly divided into two groups (n = 40 canals) and numbered in order of instrumentation.

Group 1: the canals were instrumented with WaveOne Gold Primary (025/0.7). The file was used in a programmed reciprocating motion generated by the WaveOne motor (Dentsply Maillefer, Ballaigues, Switzerland) in the ‘‘WAVEONE ALL’’ mode. The instrumentation was performed by cycles in a pecking motion (amplitude less than 3 mm) according to manufacturer’s instructions. The canal was then irrigated with 0.5 ml 96% alcohol, the patency checked with a #10 K-file (15 mm), irrigated with 0.5ml alcohol and the flutes of the file were cleaned. This procedure was repeated until the working length was reached with free rotation of the file into the canals. Finally, was performed a final irrigation with 1ml of alcohol and paper cones were used to dry the canals.

Group 2: the simulated canals were instrumented using ProTaper Next system, with files X1 (017/04) follow by X2 (025/06) in all WL. The files were operated on the WaveOne motor (Dentsply Maillefer, Ballaigues, Switzerland) with a rotational speed of 300 rpm and 4 Ncm of torque, according to the manufacturer’s instructions. A similar procedure to that of group 1 was followed. The instrumentation was performed in cycles, advancing 2-3 mm at a time in the canal. Patency was checked with the #10 K-file and irrigation with 0.5ml of alcohol was performed between the use of the files. Final irrigation with 1 ml of alcohol was accomplished after the instrumentation with the X2 file was completed. Paper cones were used to dry the canals.

The active preparation time was measured in seconds with the assistance of a digital timer. The time devoted to the irrigation process, change and cleaning of the instruments was not accounted, only the time that instrument was working on canal was recorded. In the first group was counted the instrumentation time of ProGlider and WaveOne Gold Primary. In the second group was registered the time obtained by ProGlider, X1 and X2.

The number of passes (cycles) with the file in each canal was also counted for each system (not including ProGlider file). The instrumentation technique was followed according to the manufacturer’s instructions, and one pass was counted for each “instrumentation cycle”, which included the insertion of the rotating instrument in the canals with 2-3 mm of passive penetration. These cycles were repeated and recorded until WL was reached.

-Statistical Analysis

The statistical analysis was obtained using the IBM ® SPSS ® Statistics version 25.0.0 software.

According to variables involved, the analysis consisted of:

• In the descriptive study - quantitative variables (profile graphs and summary statistics Tables).

•In the comparative study - differences in instrumentation time and number of file passages between the two instrumentation techniques (WOG vs. PTN) were evaluated by the Mann - Whitney and Kruskal-Wallis tests.

## Results

 There were no statistically significant differences in instrumentation time between the two techniques (*p*> 0.05, Table 1).

**Table 1:**
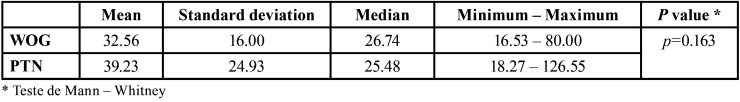
Instrumentation time for both techniques (WOG – WaveOne Gold; PTN – ProTaper Next).

**Figure 1:**
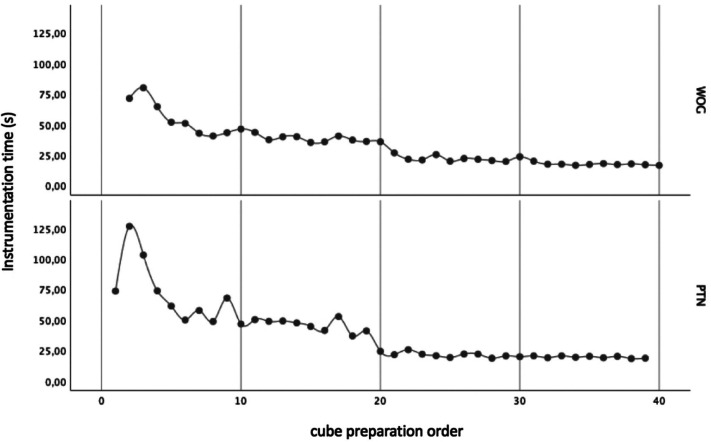
Instrumentation time profile graph according to cube preparation order in each instrumentation technique (WOG - WaveOne Gold; PTN - ProTaper Next).

Dividing the resin cubes in order of preparation (from 1st to 40th) into 4 groups (n = 10), in each technique, it was verified that there were statistically significant differences between the groups (*p* <0.05), regarding the instrumentation time (Table 2, Fig. 1).

**Table 2:**
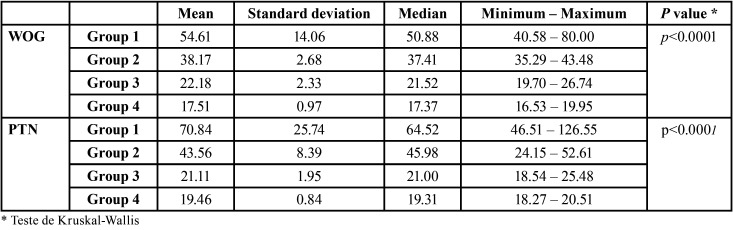
Instrumentation time, by group, for each instrumentation technique (WOG – WaveOne Gold; PTN – ProTaper Next).

**Figure 2:**
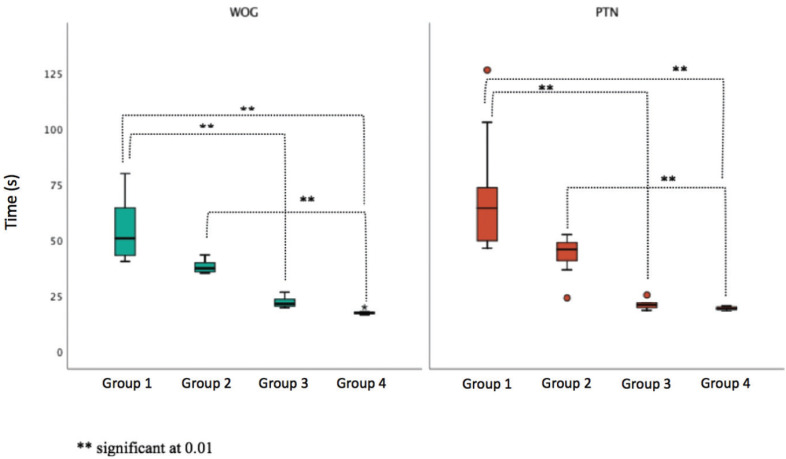
Comparisons of instrumentation time by group in each technique (WOG - WaveOne Gold; PTN - ProTaper Next).

The multiple comparison tests between group pairs showed statistically significant differences between groups 1‡3, 1‡4 and 2‡4 regardless of the technique used (Fig. 2).

Regarding the number of instrument passes (number of cycles) there were statistically significant differences between the two instrumentation techniques (*p* <0.05, Table 3).

Dividing the resin cubes in order of preparation into 4 groups, it was showed statistically significant differences between groups in relation to cycle numbers (*p* <0.05) (Table 4).

**Table 3:**
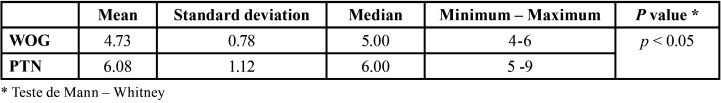
Number of instrument passes for each technique (WOG - WaveOne Gold; PTN - ProTaper Next).

**Table 4:**
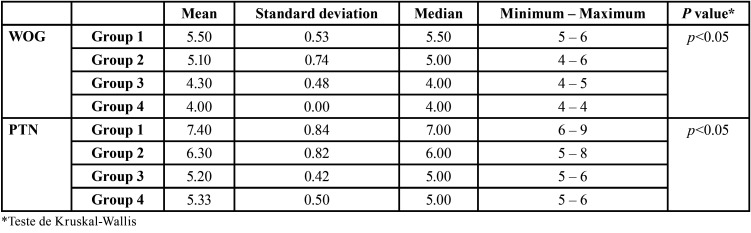
Number of instrument passes by group for each technique (WOG - WaveOne Gold; PTN - ProTaper Next).

**Figure 3:**
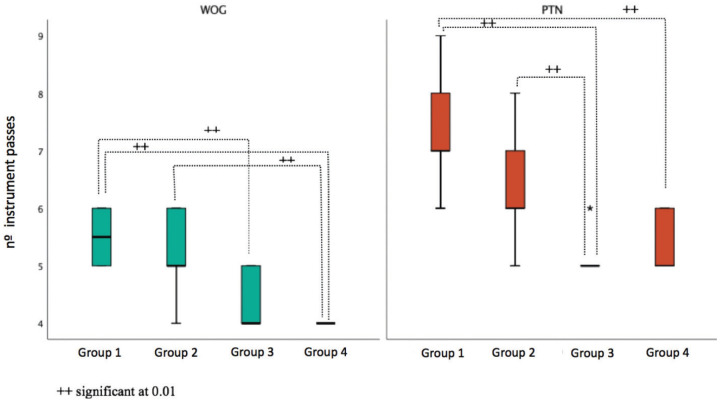
Multiple comparisons of the number of instrument passes by groups in each technique (WOG - WaveOne Gold; PTN - ProTaper Next).

The multiple comparison tests between group pairs showed statistically significant differences between group 1‡3, 1‡4 and 2‡4 g for the WOG technique. And between group 1‡3, 1‡4 and 2‡3 for PTN technique (Fig. 3). The number of passes decreased from group 1 to group 4 in both techniques.

## Discussion

The aim of this study was to compare two rotary systems (WaveOne Gold e ProTaper Next) regarding the time spent preparing simulated canals, performed by a student without experience in mechanized instrumentation.

Preparation time is influenced by the technique, number of instruments used, the operator experience and the study methodology (7,8). In the present study although the mean preparation time with WOG was shorter than PTN, there was no statistically significant difference in instrumentation time between the continuous rotation technique (PTN) and reciprocating motion (WOG) (Table 1). However, other authors associate a reciprocating movement with faster preparation (8,9). Also has been reported faster preparation with WO by experienced operator when compared to inexperienced (6,10). Cassimiro *et al.* (11) demonstrated that even when only the active instrumentation time is evaluated, the differences between WOG and PTN are significant. It should be noted that in this study were used lower incisors with straight canals. We speculated that in curved and thin canals, as in the present study, the instrumentation time with the WOG approached the time of PTN due to a greater complexity and difficulty in instrumentation requiring further number of passes with WOG.

Like other authors (12) and according to the manufacturer’s recommendation a glide path was created with the Proglider file prior to preparation with WOG or PTN. The previous use of Proglider reduces the torsional stress of rotating NiTi file, decreasing the potential risk of torsional fracture and increasing the life span of the files. This is also contributed to a lack of relevant errors in the shape of the prepared canals (12,13).

The mean preparation time with WOG was similar (10) or lower (6,14) than that reported in other studies with inexperienced operators using WaveOne. Although Goldberg *et al.* (6) study included the time for irrigation and cleaning the instruments, no previous glide path was created, and each canal was instrumented by a different student. In contrast in the present study was recorded only the active instrumentation time with the ProGlider file and WOG. The creation of a glide path decreases the preparation time of WO in inexperienced operators (10) which may justify a shorter time spent by the two instruments compared to Goldberg *et al.* study (6).

The introduction of the rotary instrumentation in undergraduate teaching has been proposed, in order to achieve a more predicTable technical quality of root canal treatment (15). In this study only one operator (an inexperienced student) prepared all the canals which might explain the decreasing average preparation time due to the learning curve that took place throughout this research. The use of NiTi rotary instruments requires a gradual learning evolution, given that the use of a hand piece for powering the instruments causes a decrease in operator tactile sensitivity on dentin walls compared to manual instrumentation (16).

The evolution of the instrumentation time according to the number of instrumented canals for both techniques is shown in Figure 1. Statistically significant differences were found between the preparation time of the first 20 cubes and the following (Table 2; Fig. 2) regardless of the system used. These results agree with Ya Yang *et al.* (17) who demonstrated that after one month of training with WO the instrumentation time of an inexperienced operator was comparable to the value obtained by an experienced operator.

For both systems the instrumentation time of the last 20 cubes decreased significantly compared to the first ones becoming comparable to the time spent by an experienced operator (10,11,18).

Has been reported that inexperienced operators take less preparation time and better performance with reciprocating systems (14,19). In addition, the use of a single-file technique reduces de risk of cross infection (20). Shorter instrumentation time reduces chair time, decreases apical extrusion of debris (21) and reduces the risk of instrument fracture, especially in posterior teeth with complex root canal anatomy (22).

Regarding the number of passages or instrumentation cycles, the PTN group presented a significantly higher number than the WOG group (Table 3), an expected result, since WOG is a single file system. However, as more canals were instrumented there was a statistically significant decrease in the number of passages (Table 4, Fig. 3). This was reflected in a decrease in instrumentation time related to better handling of both techniques. The average number of passages in the WOG group were lower than those reported by inexperienced operators, approaching the value obtained by an experienced operator with WO (6). These results may be justified by the difference between the alloys of the instruments.

To our knowledge, there are few studies evaluating the effective instrumentation time with WOG or PTN in inexperienced operators, moreover different methodologies make unable to make a direct comparison with our results (23). The use of a single operator reduced inter-operator variability, however, the conclusions presented cannot be extrapolated directly to all potential users of the evaluated techniques.

## Conclusions

Under de condition of this study both WOG and PTN instrumentation systems demonstrated predictability in preparation time, allowing a quick and easy learning, even for an undergraduate student with no prior experience using rotary systems.
